# Comparative Study Between Functional MR Urography and Renal Scintigraphy to Evaluate Drainage Curves and Split Renal Function in Children With Congenital Anomalies of Kidney and Urinary Tract (CAKUT)

**DOI:** 10.3389/fped.2019.00527

**Published:** 2020-01-28

**Authors:** Maria Beatrice Damasio, Monica Bodria, Michael Dolores, Emmanuel Durand, Fiammetta Sertorio, Michela C. Y. Wong, Jean-Nicolas Dacher, Adnan Hassani, Angela Pistorio, Girolamo Mattioli, Gianmichele Magnano, Pierre H. Vivier

**Affiliations:** ^1^Radiology Department, IRCCS Istituto Giannina Gaslini, Genoa, Italy; ^2^Nephrology and Renal Transplantation Department, IRCCS Istituto Giannina Gaslini, Genoa, Italy; ^3^CHU C. Nicolle, Service de Radiopédiatrie, Rouen University Hospital, Rouen, France; ^4^Department of Nuclear Medicine, University of Strasbourg ICube (UMR7537), Strasbourg, France; ^5^Department of Health Sciences, Radiology Department, University of Genoa, Genoa, Italy; ^6^Pediatric Surgery Department, IRCCS Istituto Giannina Gaslini, Genoa, Italy; ^7^Department of Neuroscience, Rehabilitation, Ophthalmology, Genetics and Maternal and Child Science, School of Medical and Pharmaceutical Sciences, University of Genoa, Genoa, Italy; ^8^Epidemiology, Biostatistics and Committees Unit, IRCCS Istituto Giannina Gaslini, Genoa, Italy; ^9^Ramsay - Générale de Santé, HôpitalPrivé de l'Estuaire, Radiology, Le Havre, France

**Keywords:** obstructive uropathy, dynamic renal scintigraphy, functional magnetic resonance urography, congenital anomalies of the kidney and urinary tract, CAKUT

## Abstract

**Background:** Obstructive congenital anomalies of the kidney and urinary tract have a high risk of kidney failure if not surgically corrected. Dynamic renal scintigraphy is the gold standard technique to evaluate drainage curves and split renal function (SRF).

**Objectives:** To compare functional magnetic resonance (MR) urography with dynamic renal scintigraphy in measuring volumetric SRF and in the classification of drainage curves in patients with congenital anomalies of the kidney and urinary tract.

**Materials and Methods:** We retrospectively collected patients with hydroureteronephrosis or pelvicalyceal dilatation at renal ultrasound, who underwent both functional MR urography and dynamic renal scintigraphy (DRS) within 6 months. DRS studies were evaluated by a single nuclear medicine physician with a double reading. Functional MR urography renograms were blind evaluated twice by two radiologists. The functional MR urographyintra- and inter-reading agreements as well as the agreement between the two imaging techniques were calculated. SRF was evaluated by Area Under the Curve and Rutland-Patlak methods. Drainage curves were classified as normal, borderline or accumulation patterns by both the techniques.

**Results:** Fifty-two children were studied, 14 with bilateral involvement. A total of 104 kidney-urinary tracts were considered: 38 normal and 66 dilated. Considering Area Under the Curve and Rutland-Patlak for SRF, the intra- and inter-reader agreements of functional MR urography had excellent and good results, respectively, and the two techniques demonstrated a good concordance (r2: 67% for Area Under the Curve and 72% for Rutland-Patlak). Considering drainage curves, the inter-readers agreement for functional MR urography and the concordance between the two techniques were moderate (Cohen's k, respectively, 55.7 and 56.3%).

**Conclusions:** According to our results, there are no significant differences between functional MR urography and DRS in measuring volumetric SRF and in the classification of drainage curves in patients with congenital anomalies of the kidney and urinary tract.

## Introduction

Congenital obstructive uropathy is the most frequent anomaly of the urinary tract occurring in up to 2% of normal pregnancies (1, 2). The prognosis is good in most cases but 25% of patients show persistent severe hydronephrosis with a high risk of irreversible reduction of kidney function if not surgically corrected (2, 3). Dynamic renal scintigraphy (DRS) is the current gold standard technique to evaluate obstruction (by drainage curve) and to measure the split renal function (SRF) between the two kidneys in children and young adults (4, 5), driving therapeutic decisions. Functional Magnetic Resonance (MR) urography has been recently proposed by many study groups as an alternative technique to evaluate the drainage curve and SRF in obstructive uropathy in the last decade (6–10). Currently Functional MR urography is becoming established within clinical practice as it provides a depiction of deep anatomical details thanks to its intrinsically high soft-tissue contrast (11–13). This is extremely useful on complex phenotypes such as congenital anomalies of the kidney and urinary tract (CAKUT) spectrum diseases, with the further benefit of eliminating the exposure to ionizing radiation. The use of high-resolution techniques may be extremely important in subjects affected by CAKUT, in whom the embryology and genetic background complexity determines the drastic anatomical differences within each single case, even when from the same family (14). Moreover, the clinical validation of functional MR urography for the evaluation of uretero-pelvic junction obstruction has been recently published and could be introduced into clinical practice (15).

Comparative studies on volumetric SRF evaluation by DRS and functional MR urography have shown a good correlation between the two techniques, where functional MR urography offers the advantage of calculating the volume of enhanced renal parenchyma (16–21). Although these provide evidence for a comprehensive multicenter comparative study on different CAKUT phenotypes which calculate the volumetric SRF using the Area Under the Curve, the Rutland-Patlak method and the volume proportion expressed as a percentage together with the comparative evaluation of drainage curves is lacking. This may limit the routinely use of functional MR urography into clinical practice.

The aim of this study was to compare functional MR urography with DRS in measuring volumetric SRF and in the classification of drainage curves in patients affected by different obstructive CAKUT phenotypes.

## Materials and Methods

We retrospectively selected patients with hydroureteronephrosis or pelvicalyceal dilatation at renal ultrasound, who underwent both functional MR urography and DRS within 6 months without any urinary tract infection or surgical intervention in-between. The cohort consisted of patients studied between 2010 and 2014 in two different Pediatric Nephrology centers. No clinical cases studied after 2014 were eligible, mostly because a concomitant functional MR urography and DRS evaluation were not available.

Pelvicalyceal dilatation was defined as antero-posterior diameter equal or superior to 10 mm (22) at renal ultrasound and confirmed at MR. All patients had normal renal function at recruitment (eGFR > 90 ml/min/1.73 m^2^ calculated by Schwartz equation in children and CKD-EPI equation in young adult) (23, 24). The different anatomical phenotypes included in the sample are reported in Table 1. The anatomical classification was derived from MR morphological studies following the International Statistical Classification of Disease and Related Health Problems, Tenth Revision (ICD-10). All cases were first examined by renal ultrasound as routinely recommended. Morphological MR finding confirmed all the renal ultrasound diagnosis. The normal kidneys and urinary tracts of patients who carried malformation on one side only were used as controls. Complex CAKUT (cases with associated congenital anomalies out of obstructive phenotype such as vescicoureteral reflux) were excluded to better interpret obstructive uropathy. Vesico-ureteral reflux and posterior urethral valves were excluded in all the patients by a voiding cystourethrography.

**Table 1:** Allocation of different anatomical phenotypes reported in number of kidney and urinary tract by side.

**Kidney and urinary tract: phenotype and number of cases by side**	
**Right side**	**Left side**
Normalanatomy 25	Normalanatomy 13
Uretero-pelvicjunctionobstruction 14	Uretero-pelvicjunctionobstruction 26
Primitive megaureter 8	Primitive megaureter 10
Mid ureteral stenosis 1	Mid ureteral stenosis 1
Other 4	Other 2

The same protocol has been used on the same MR scanner (1.5 Tesla MR scanner AchievaIntera; Philips Medical System Best, The Netherlands) with cardiac or abdominal cardiac phased-array surface coils depending on a patient's age. The same DRS protocol has been used in both Institutes.

All functional MR urography imaging studies were independently and blind evaluated twice by two high level trained Pediatric Radiologists of the two research Centers, with a total of four readings, with a temporal interval of at least 6 months between both analyses of each reader. The scintigraphic studies were evaluated by a single Nuclear Medicine Physician with double reading.

As the study was observational and retrospective in design and did not examine patients' personal information, ethical committee approval was not required.

### Dynamic Renal Scintigraphy

Patients were given 10–20 mL/Kg of water orally 30–40 min before the procedure. Posterior dynamic acquisition was performed after intravenous injection of 3.7 MBq/kg of body weight of 99 mTc mertiatide and 1 mg/kg of body weight of furosemide (with a maximum of 20 mg). Images were processed by an independent senior nuclear medicine physician by a home-made software programmed with Matlab® (Mathworks, Natick, Mass). Regions of interest were manually drawn on kidneys, heart and C-shaped perirenal background. Relative function was determined using the Patlak-Rutland method, or the Area Under the Curve method in studies in which the cardiac curve did not meet enough quality, according to international consensus recommendation (19). Drainage was quantitatively assessed by NORA (normalized residual activity), Renal Output efficiency and Tmax. The operator then classified the drainage as normal, borderline or poor.

### Functional MR Urography

Functional MR urography were performed following the protocol by Vivier et al. (7) and analyzed using the freely available ImageJ MRU software (8).

The management of the sedation step during non-collaborating patient preparation was modestly differentiated in the two centers. Center 2 totally followed the original protocol (7) applying the oral sedation by hydroxyzine in subjects older than 6 months of age, Center 1 used inhaled sedation (by sevoflurane gas) under anesthesiologist control. No differences in preparation were used in children younger than 6 months in the two group, where the feed and wrap technique (25, 26) was confirmed to be efficient.

**Figure 1:**
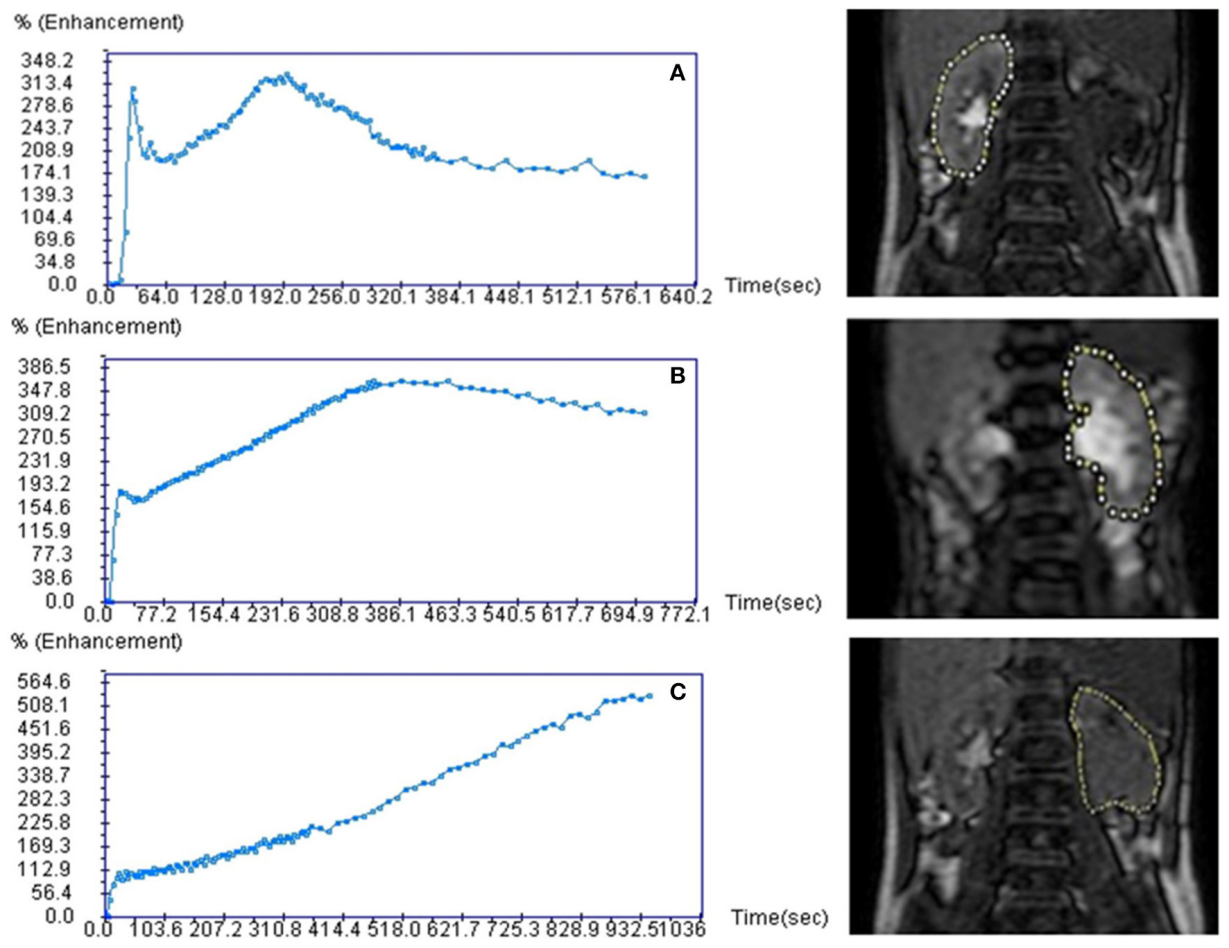
Functional magnetic resonance urography drainage curves (fMRU-DC) classification. Curves are classified under a normal, borderline and accumulation patterns. Normal fMRU-DC shows a decreasing of the signal intensity after the filtration peak **(A)**; borderline fMRU-DC remains stable after the filtration peak, drawing a plateau **(B)**; accumulation fMRU-DC shows an ever-increasing signal intensity **(C)**.

The functional MR urography evaluation was based on two sequences. The first was a single-shot coronal T2-weighted sequence (slice thickness 4 mm, gap zero) with a volumetric coverage of the kidney for volume measurement by a dedicated 3D segmentation algorithm (8) which was previously validated by an *in vitro* study (27). The second sequence called T1-weighted 3D gradient echo was a dynamic contrast-enhanced renographic sequence covering the aorta and the kidneys and it was acquired after injection of a micro-bolus 0.05 mmol/kg of gadoteratomeglumin elution of 5 s for the first 5 min, then 30 s over the next 5 min. The second sequence was used to assess the SRF and drainage curve in order to evaluate potential obstruction. SRF of renal pixels were evaluated by two methods: (A) Area Under the Curve-obtained by placing a region of interest on renal parenchyma on a single slice displaying the most of renal tissue, (B) the Rutland-Patlak method obtained by placing an additional region of interest over the supra-renal-aorta. Renal volumes and pixel-based SRF were combined to obtain the volumetric SRF. drainage curve was obtained by placing a region of interest including renal parenchyma and pelvis as DRS. Based on O'Reilly DRS-drainage curve classification (5) and on Vivier's paper (8), the authors adopted a common drainage curve classification system: normal, borderline and accumulation patterns (Figure 1). Normal functional MR urography-drainage curve exhibits a decrease in the signal intensity after the filtration peak (Figure 1A); borderline functional MR urography-drainage curve remains stable after the filtration peak, drawing a plateau (Figure 1B); accumulation functional MR urography-drainage curve shows an ever-increasing signal intensity (Figure 1C).

The reproducibility of the intra-reader was assessed for volumetric SRF and drainage curve lectures in both the two techniques. Inter-reader reproducibility of functional MR urography renal volume calculation, volumetric SRF and drainage curve were evaluated.

Since significant motion artifacts may confound or preclude interpretation leading to diagnostic errors, we removed all the imaging studies that could not be evaluated.

### Statistical Analysis

The normality distribution of the data was evaluated using the D'Agostino-Pearson test. A correlation coefficient was used to identify intra and inter-reader reproducibility among the different readers and -reader reproducibility among the two methods for all the parameters considered. A non-parametric Friedmand test for parried samples and the Kruskall-Wallis test for unpaired samples were, respectively, used to assess differences between Area Under the Curve or Rutl and-Patlak parameters on technical replicates and methods. The Bland-Altman plot was used to quickly visualize the correspondence between the two techniques. The limits of the correspondence were calculated using mean ± 1.96 × standard deviation and their confidence interval. Spearman's correlation coefficients were calculated between DRS SRF and functional MR urography SRF considering, respectively, kidney volumes and Area Under the Curve and Patlak volumetric SRF. We separately considered volume evaluation in normal and pathological kidneys and urinary tracts for each reader and between the two readers.

The concordance of functional MR urography-drainage curve classification into the three curve categories between reader 1 and reader 2 were analyzed by the Cohen's kappa coefficient.

A contingency table was used to determinate the capacity of the functional MR urography for the assessment of renal drainage outcomes in comparison to DRS for a single reader since the functional MR urography-drainage curve classification concordance was acceptable.

The reader evaluation of functional MR urography-drainage curve compared to DRS-drainage curve for each patient was reported. Diagnostic effectiveness (proportion of patients correctly categorized by the functional MR urography) and misclassification rate (proportion of patients who were incorrectly classified by the functional MR urography) were calculated from the table; the validity and reliability of concordance were calculated using the Cohen's kappa coefficient and their concordance were indicated using Cohen's kappa value. Kappa values can be interpreted as follows: <0.01 null; 0.01–0.20, slight; 0.21–0.40, fair; 0.41–0.60, moderate; 0.61–0.80, substantial; and 0.81–0.99, almost perfect agreement (28).

For all statistical analysis, the significance was *p* < 0.05 for the two tails. All the analysis was performed using software package R last version available at the time of experiments.

## Results

Fifty-two children were included, 14 with bilateral involvement; 13 females, 39 males; age range 0 months−19 years, median age 1 year old (only 2 cases older than 15 years, 17 cases younger than 1 year). Imaging studies were acquired from 2010 to 2014 in two centers: Center 1 (16 cases) and Center 2 (36 cases). Only 6 of 52 (11.5%) cases had a longer interval between the two radiologic tests and half of them were 5 years old or older. One patient had an interval between tests of 5 months; one patient of 4 months; four patients of 3 months; globally the cohort had an interval between the two tests equal or minor of 2 months. A total of 104 kidney-urinary tracts were considered: 38 normal and 66 dilated. The normal kidneys and urinary tracts were used as controls. Eight subjects were excluded because DRS (six cases) or functional MR urography (two cases) data to derive volumetric SRF and drainage curve were unavailable. Thirty-two/38 normal kidneys and 56/66 pathological kidneys had both scintigraphic and functional MR urography and drainage data suitable for the analysis. DRS exams were limited in few cases (six cases) by a late acquisition resulting in poor arterial input function, while 16 kidney-urinary tracts were not evaluable for volumetric SRF and/or drainage curve for patient movements or contrast medium extravasation during functional MR urography. Three/52 patients underwent sedation for functional MR urography execution. No adverse effects were observed during functional MR urography and DRS procedures.

### Evaluation of Functional MR Urography and DRS Equivalence in Measuring Volumetric SRF

The intra-reader repeatability in assessing renal volume in normal kidneys and urinary tracts by functional MR urography was 0.97 for reader 1 and 0.99 for reader 2, whereas it was 0.95 and 0.96, respectively, in pathological kidneys and urinary tracts. The inter-reader repeatability also resulted with high r2 Spearman value (0.93 in normal kidneys and urinary tracts and 0.95 in pathological ones).

### Volumetric SRF Intra-reader Repeatability

For each reader, the high correlation coefficient indicated an excellent intra-observer repeatability for Area Under the Curve and Rutland-Patlak measurements in volumetric SRF. Scintigraphic method had a higher correlation coefficient than functional MR urography method (Table 2). No significant differences were determined when the values of technical replicates were compared.

**Table 2:** Results of intra and inter-reader correlation coefficients and bias analysis for each reader and method of measurement on area under the curve and Patlak parameters.

		**Area under the curve%**	**Patlak%**
fMRU Intra-reader (reader 1)	R^2^	0.61	0.63
fMRU Intra-reader (reader 2)	R^2^	0.80	0.82
fMRU Inter-reader	R^2^	0.72	0.74
DRS Intra-reader	R^2^	0.98	0.94
Inter-reader DRS vs. fMRU	R^2^	0.67	0.72
DRS vs. fMRU	P	0.92	0.96

*fMRU, functional magnetic resonance urography; DRS, dynamic renal scintigraphy*.

### Volumetric SRF Inter-reader Repeatability

The correlation coefficient obtained between the two methods for each parameter (Area Under the Curve and Patlak) indicated a good inter-reader repeatability (Table 2). In addition, no statistically significant differences were determined when we compared the measurements of the two methods.

### Volumetric SRF DRS vs. Functional MR Urography Concordance

**Figure 2:**
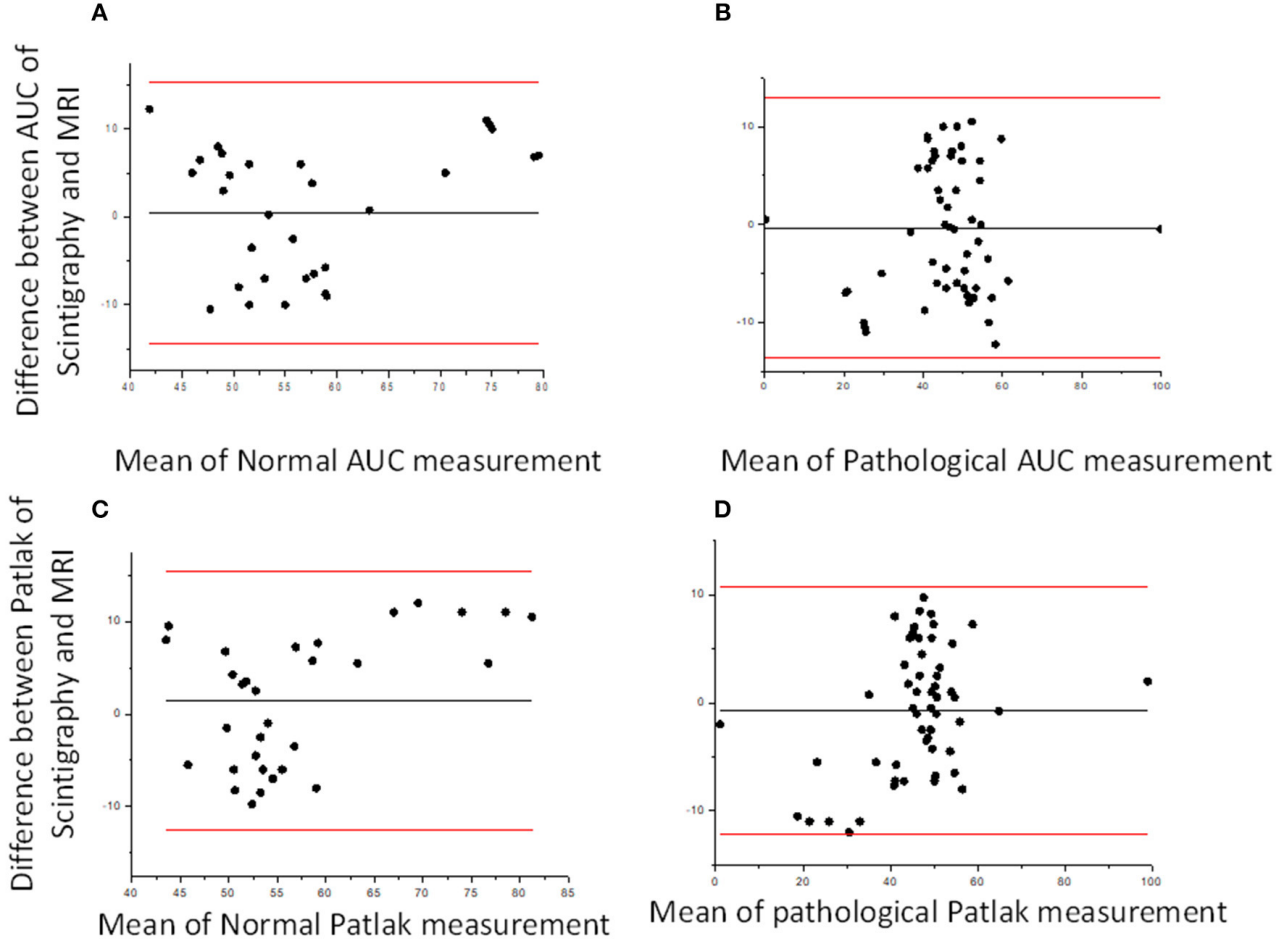
Bland–Altman plots. Bland–Altman plot comparing DRS and functional MR urography measurement in Area Under the Curve (AUC) **(A,B)** and Patlak **(C,D)** for normal and pathological kidney, respectively. The solid lines represent, respectively, mean, upper and lower limits of the agreement for each parameter considered.

Considering the good functional MR urography inter-reader repeatability, the concordance between the two techniques in evaluating volumetric SRF was based on the Reader 2 results. The two methods demonstrated a good concordance between the measurement of Area Under the Curve and Rutland-Patlak parameters in volumetric SRF. The mean difference, their agreement limits and confidence interval were reported in Table 3. These were depicted in the Bland–Altman plot which indicates that both methods have good correspondence (Figure 2). No differences in correspondence were determined depending on the parameters considered.

**Table 3:** Mean differences, agreement limits and confidence interval of Area Under the Curve and Rutland-Patlak parameters between functional magnetic resonance urography and dynamic renal scintigraphy in normal and pathological kidneys and urinary tracts.

**Normal Kidney**		**Area under the curve%**	**Patlak%**
	Means	0.50	1.51
	Standard deviation	7.57	7.13
Agreement limit	Upper	15.34	15.49
	Lower	−14.35	−12.47
**Pathological Kidney**		**AUC%**	**Patlak%**
	Means	−0.37	−0.76
	Standard deviation	6.78	5.84
Agreement Limit	Upper	12.91	10.69
	Lower	−13.66	−12.21

Considering the DRS SRF correlation, respectively, with volume percentage and volumetric SRF we found a higher correlation coefficient between DRS SRF and volumetric SRF (Spearman's correlation coefficients 0.54 with a *P* < 0.005 and 0.56 with a *P* < 0.005), both derived from Area Under the Curve and Patlak method.

### Evaluation of Functional MR Urography and DRS Equivalence in the Classification of Drainage Curves

Reader 1 and reader 2 had an observed agreement of 72% with a Cohen's Kappa 55.7% in functional MR urography-drainage curve classification.

Diagnostic effectiveness and the misclassification rate of functional MR urography-drainage curve (considering reader 2) compared to DRS-drainage curve are shown in Table 4. DRS and functional MR urography results showed a moderate level of concordance (observed agreement = 71.6%; Cohen's k = 56.3 % with 95% Confidence Interval: 41.4–71.2%). The drainage curve resulted to be normal in 32 kidneys and urinary tracts, borderline in 18 and in accumulation in 13, both by DRS-drainage curve and functional MR urography-drainage curve. Seven kidneys and urinary tracts with borderline DRS-drainage curve resulted with normal functional MR urography-drainage curve; both 1 normal DRS-drainage curve and 1 normal functional MR urography-drainage curve had the result of an accumulation pattern by means of the other technique.

**Table 4:** Diagnostic effectiveness and misclassification rate of functional magnetic resonance urography(fMRU) drainage curves compared to dynamic renal scintigraphy (DRS) drainage curves.

	**fMRU-drainage curves**			
**DRS-drainage curves**	**Normal**	**Borderline**	**Accumulation**	**Total**
Normal	32	2	1	35
Borderline	7	18	5	30
Accumulation	1	9	13	23
Total	40	29	19	88

## Discussion

Congenital anomalies of the kidneys and upper urinary tract encompass a wide spectrum of anomalies varying from asymptomatic to life threatening conditions. Hydronephrosis is the major congenital malformation detected by ultrasound in prenatal setting (1). Most of these cases evolve toward a spontaneous resolution in the first year of life, while a minority will develop a real obstruction (2). Considering the urological system plasticity, these children may require multiple functional evaluations during their growth in order to follow CAKUT evolution. In the case of persistent urinary tract dilatation, a functional test may be necessary also after surgery. Moreover, the same child is often studied with other radiological procedures (i.e., voiding cystourethrography) to exclude the concomitant presence of other anatomical anomalies that may be responsible for worse renal outcome (14, 29), with a risk of exposure to ionizing radiation.

Functional MR urography emerged as a powerful modality for the assessment of pediatric nephropathy. It is a “one-stop-shop” technique (30) able to give high resolution multiplanar anatomical details on the genito-urinary system and functional results. This is extremely useful in pediatrics, where highly complex malformations benefit of a precise definition in a unique session without radiation exposure (3, 14).

Despite the growing role of functional MR into clinical setting, DRS continues to be considered as the gold standard technique to functionally evaluate hydroureteronephrosis and especially to drive therapeutic decisions (4, 5) since few comprehensive studies have been published on the comparison between the two techniques in different CAKUT phenotypes. Moreover, fMRU has not yet been used as a technique for grading obstructive uropathy where DRS remains the gold standard (4, 10).

A major limitation of MR urography is that SRF is comparatively evaluated considering both kidneys. In case of the diminished function of one kidney, results may be misleading and complementary scintigraphic test may be necessary. In our study, the comparison between functional MR urography and DRS in measuring volumetric SRF and drainage curves in our selected patients with CAKUT, contribute to confirming the equivalence between the two techniques and between different centers. Area Under the Curve and Rutland-Patlak methods were applied to derive volumetric SRF as described by Vivier (12). Inter-reader repeatability in measuring volumetric SRF between the two centers and between the two techniques showed a good correlation. The accuracy and repeatability of the 3D segmentation algorithm based on the belief function theory for calculating renal volumes were shown by Vivier et al. as part of ImageJ plug-in software for functional MR urography evaluation (27). The use of functional MR urography in determining kidney volume is already accepted as the gold standard in clinical practice regarding complex anatomy architecture cases such as autosomal dominant polycystic kidney disease (31, 32). Contrary to recently reported results (21), we found that the absolute difference in volumetric SRF between functional MR urography and DRS did not exceed the accepted tolerance threshold of 5%. The major difference between the protocol adopted in this study and the one from other groups (21, 33) is the time and modality of furosemide and Gadolinium-based Contrast Agents administration: while we use the F-0 technique and a micro-bolus of Gadolinium-based Contrast Agents (7) injection at higher flow rate, other groups adopt the F-15 method (34) with slow Gadolinium-based Contrast Agents injection. We suppose that the use of ImageJ MRU plug-in protocol associated with the original method of F-0 technique has better correlation with volumetric SRF calculated by DRS and this may explain differences from previous reported data. We underline that results on the evaluation of SRF may be correlated to normal renal function of all cases.

The comparison between functional MR urography and DRS in measuring obstruction can be evaluated by functional MR urography and DRS renal transit time (21) or by time-intensity-drainage curve morphology according to O'Reilly's classification (5, 35). In order to simplify the comparison between the two imaging techniques, we introduced a common drainage curve classification system based on normal, borderline and accumulation curves (Figure 1). We considered the three patterns of functional MR urography-drainage curve to DRS-drainage curve grading system including normal and pathological kidneys and urinary tracts.

In our series, within the 23 kidneys and urinary tracts with accumulation DRS-drainage curve, nine resulted borderline and one normal by functional MR urography. Within the 30 borderline DRS-drainage curve, seven resulted normal and five with accumulation by functional MR urography, differently from previous results (6, 17). This difference between the two techniques in evaluating obstruction both in accumulation and borderline patterns may be secondary to the different diuretic administration protocol: functional MR urographyis a F0 (7) while DRS is a F20 technique (4). The best technique between DRS and functional MR urography in evaluating obstructive uropathy in accumulation renal curves would require an animal study with a Whitaker test, or a long-time follow-up along with surgical findings. Anyway, a previous study by our group comparing open surgery with MR urography findings demonstrated high level of concordance (15).

Our study shows that DRS and functional MR urography have no differences in terms of safety and feasibility. We consider functional MR urography as a safe, radiation-free technique to detect and to evaluate obstructive uropathy in children and young adults (36). It is necessary to underline the recent alert on the use of Gadolinium-based Contrast Agents particularly related to its possible deposition in central nervous system tissues (37). However, the macrocyclic are considered safer than the linear gadolinium-based contrast agents (38, 39) furthermore a micro-bolus with a low amount of contrast injection is adopted. Moreover, in non-collaborating children, when the feed and wrap technique is not applicable, sedation may be required; in the selected cohort only 3/52 children (5–6%) underwent sedation.

The use of functional MR urography is still hampered by its post-acquisition analysis process that requires about 45 min per patient in comparison with <10 min with DRS. A more automatized MR post-processing tool might be of great interest to speed up this duration and may probably improve the reproducibility of SRF measurement.

The study has major limitations. It is a retrospective study and the cohort consist of patients studied between 2010 and 2014. No clinical cases studied after 2014 were eligible mostly because a concomitant functional MR urography and DRS evaluation were not available. The scintigraphic studies were evaluated only by a single operator with double reading, whereas functional MR urography exams were evaluated by two Radiologists. This not only introduced a bias in the analysis, but did not allow to calculate the inter-reader agreement for DRS. As shown in Table 2 the intra-reader agreement of DRS shows a strong concordance between different lectures by the same reader. This may be supported by the more automatic analysis of DRS data than the one of functional MR urography where manual segmentation and ROI definition is required. A minor limitation of the study is the presence of artifacts in some functional MR urography and DRS acquisitions which reduced the sample size.

Despite the limitations described, the results from the selected cohort analyzed deserve scientific consideration. Performing a new comparative study between MR and DRS would be difficult for high costs and ethically objectable because of data already available in literature and the result of this study that confirm the role of functional MR urography in CAKUT patients with OU.

A major concerns regarding the wide use of functional MR urography in clinical practice remain the limited access to MR, especially in peripheral centers, its cost and the post-processing analysis which requires time and dedicated Pediatric Radiologists. However, the advantage of MR in reducing exposure to ionizing radiation and in depicting fine anatomical details, which is extremely useful for surgical approach, makes this technique strongly recommended in CAKUT. Complex CAKUT cases should be addressed to a second or third level center for a three-dimensional diagnostic assessment and for the new precision medicine programs that will be offered in the next future simultaneously with knowledge from new generation data (40).

## Conclusions

According to our results, there are no significant differences between functional MR urography and DRS in the classification of drainage curves in patients with congenital anomalies of the kidney and urinary tract. DRS remains useful in evaluating quantitative total renal function and deriving the SRF mostly in patient with reduced renal function.

Functional MR urography is emerging as an excellent imaging technique able to combine detailed anatomical information together with a functional evaluation, which is extremely useful in complex congenital anomalies of the kidney and urinary tract.

Major limitations to expand the use of this technique are the lack of trained pediatric radiologists and the limited access to MR. Anyway, complex CAKUT cases should be addressed to a second or third level center for a three-dimensional diagnostic assessment.

## Data Availability Statement

The datasets generated for this study are available on request to the corresponding author.

## Ethics Statement

As the study was observational and retrospective in design and did not examine patients' personal information, ethical committee approval was not required.

## Author Contributions

MBD, MB, GMat, GMag, FS, and MW: collecting clinical data in Center 2 and analyzing the results. AP: performing statistical analysis. ED: nuclear medicine data analysis. MD, AH, PV, and J-ND: collecting clinical data in Center 1 and analyzing results.

### Conflict of Interest

The authors declare that the research was conducted in the absence of any commercial or financial relationships that could be construed as a potential conflict of interest.
